# Exploring swine oviduct anatomy through micro-computed tomography: a 3D modeling perspective

**DOI:** 10.3389/fvets.2024.1456524

**Published:** 2024-09-03

**Authors:** Ramses Belda-Perez, Costanza Cimini, Luca Valbonetti, Tiziana Orsini, Annunziata D’Elia, Roberto Massari, Carlo Di Carlo, Alessia Paradiso, Seerat Maqsood, Ferdinando Scavizzi, Marcello Raspa, Nicola Bernabò, Barbara Barboni

**Affiliations:** ^1^Department of Biosciences and Technology for Food, Agriculture and Environment, University of Teramo, Teramo, Italy; ^2^Physiology of Reproduction Group, Department of Physiology, Faculty of Veterinary Medicine, International Excellence Campus for Higher Education and Research (Campus Mare Nostrum), University of Murcia, Murcia, Spain; ^3^Institute of Biochemistry and Cell Biology (CNR-IBBC/EMMA/Infrafrontier/IMPC), National Research Council, Rome, Italy

**Keywords:** oviduct, microCT, 3D-reconstruction, swine model, utero tubal junction, ampullary-isthmic junction

## Abstract

The oviduct plays a crucial role in the reproductive process, serving as the stage for fertilization and the early stages of embryonic development. When the environment of this organ has been mimicked, it has been shown to enhance *in vitro* embryo epigenetic reprogramming and to improve the yield of the system. This study explores the anatomical intricacies of two oviduct regions, the uterotubal junction (UTJ) and the ampullary-isthmic junction (AIJ) by using micro-computed tomography (MicroCT). In this study, we have characterized and 3D-reconstructed the oviduct structure, by measuring height and width of the oviduct’s folds, along with the assessments of fractal dimension, lacunarity and shape factor. Results indicate distinct structural features in UTJ and AIJ, with UTJ displaying small, uniformly distributed folds and high lacunarity, while AIJ shows larger folds with lower lacunarity. Fractal dimension analysis reveals values for UTJ within 1.189–1.1779, while AIJ values range from 1.559–1.770, indicating differences in structural complexity between these regions. Additionally, blind sacs or crypts are observed, akin to those found in various species, suggesting potential roles in sperm sequestration or reservoir formation. These morphological differences align with functional variations and are essential for developing an accurate 3D model. In conclusion, this research provides information about the oviduct anatomy, leveraging MicroCT technology for detailed 3D reconstructions, which can significantly contribute to the understanding of geometric-morphological characteristics influencing functional traits, providing a foundation for a biomimetic oviduct-on-a-chip.

## 1 Introduction

The oviduct is the organ where the journey of life begins for all mammals. It is divided into 4 regions, the infundibulum, the ampulla, the isthmus, and the uterotubal junction. After ovulation, the infundibulum is responsible for collecting the cumulus-oocyte complex, and on the isthmus, the sperm will form the spermatic reservoir attaching to the epithelial cells, suffering changes in its membrane, and prolonging their viability. Before ovulation, spermatozoa will be released from the reservoir and they reach the ampulla, where fertilization takes place. On the other hand, the uterine-tubal junction could be considered one of the main selection barriers (1).

The epithelium of the oviduct is composed of two major types of cells: secretory and ciliated. The secretory cells are responsible for the formation of oviductal fluid (2), which plays an important role in creating an appropriate environment for the transport and nourishment of gametes, as well as in protecting the fertilized egg during its journey through the oviduct (3). Conversely, the ciliated cells possess carbohydrate residues that are recognized by lectin-like proteins on the head of spermatozoa, leading to their binding and the creation of the previously mentioned sperm reservoir (4). The oviduct offers a dynamic environment, since its cell proportion and functionality change in response to the hormonal swings that take place throughout the cycle (5, 6). For instance, during the follicular phase, ciliated cells prevail in the ampulla of the oviduct while during the luteal phase, secretory cells take the forefront in this region (7). By contrast, in other segments of the oviduct, like the isthmus, the proportion of these cell types remains relatively stable with minimal variations throughout the estrus cycle (7).

Due to the relevance of oviduct in fertilization, some authors have tried to mimic its effect in *in vitro* fertilization platforms used in artificial reproductive techniques (8, 9). Indeed, the addition to artificial environment designed to allow the fertilization could improve the system’s out, mainly reducing the epigenetic differences between the *in vivo* derived and *in vitro* produced embryos.

In that context, for instance an oviduct-on-a-chip has been recently created (9). In one hand it represents a very interesting device, but in the other one hand it ignores the architectural features of the organ. Since it has been demonstrated that there is a correlation between oviduct architecture and function (10) here we carried out a set of measures to lay the foundation to a sort of reverse engineering work. In fact, in a recent work we identified, from a selection of 3D-printing-biocompatible materials previously used in cell cultures, the one suitable for construction the model of the oviduct, evaluating its toxic effects by mean of embryo development (11). Consequently, the present work emerges as a pilot study aimed at creating an anatomically accurate-3D model of the oviduct to furtherly design a biomimetic oviduct-on-a-chip. This approach will consider the organ’s morphology, then we adopted an approach based on the use of microcomputed tomography (MicroCT) as a reliable tool for the anatomical study of oviduct: it is non-destructive technology characterized by high resolution and three-dimensional visualization capabilities, and it is able to allow sophisticated quantitative analysis and detailed 3D reconstructions.

## 2 Results

### 2.1 Utero tubal junction

**Figure 1:**
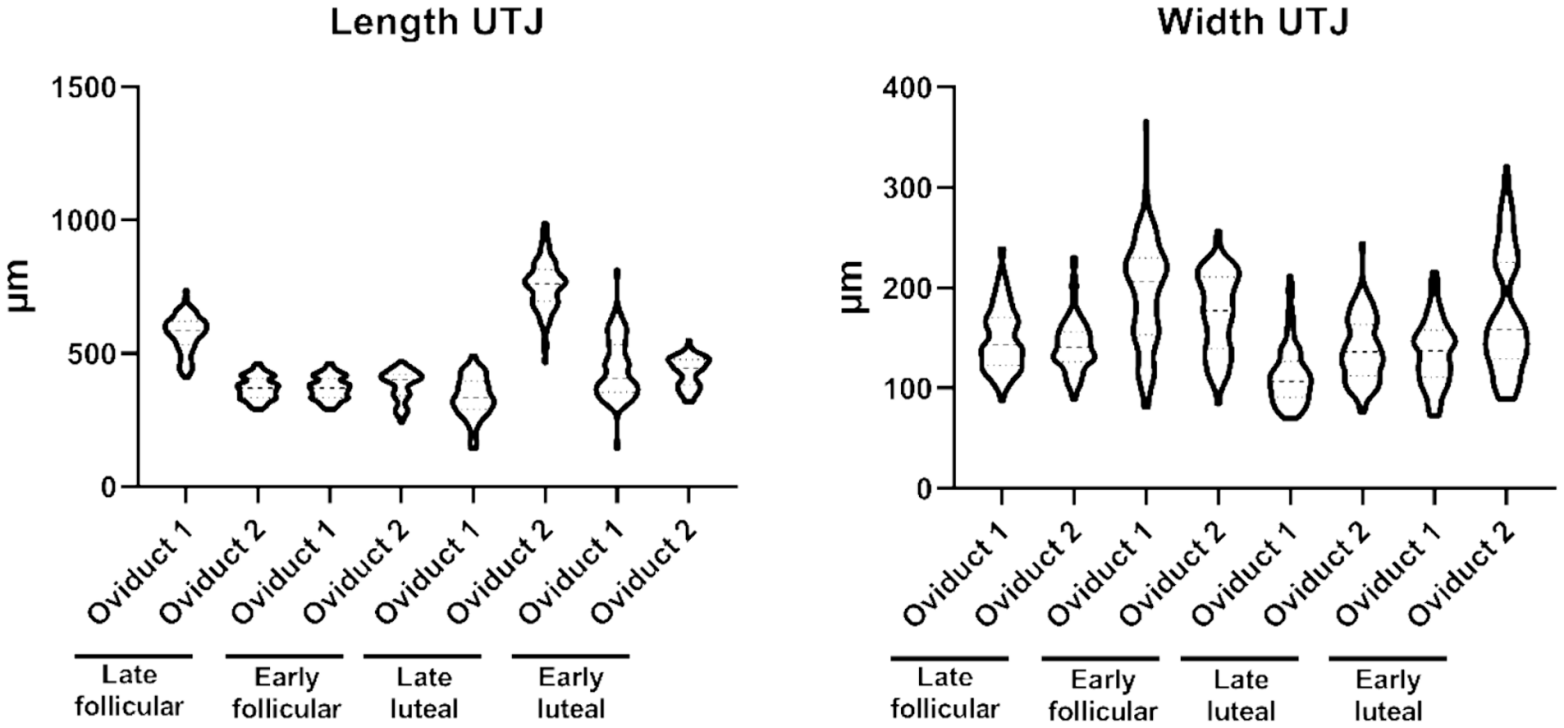
Violin plot representing the different measurement of length (left) and width (right) of the folds from the UTJ.

In this segment of the oviduct, it is noteworthy that there are small folds present, and their dimensions typically fall within the range between 144–988 μm length and 70–366 μm width (Supplementary Table S1; Figure 1). These folds do not reach a great percentage of the lumen, an observation that is supported by the high lacunarity value (Supplementary Table S1).

**Figure 2:**
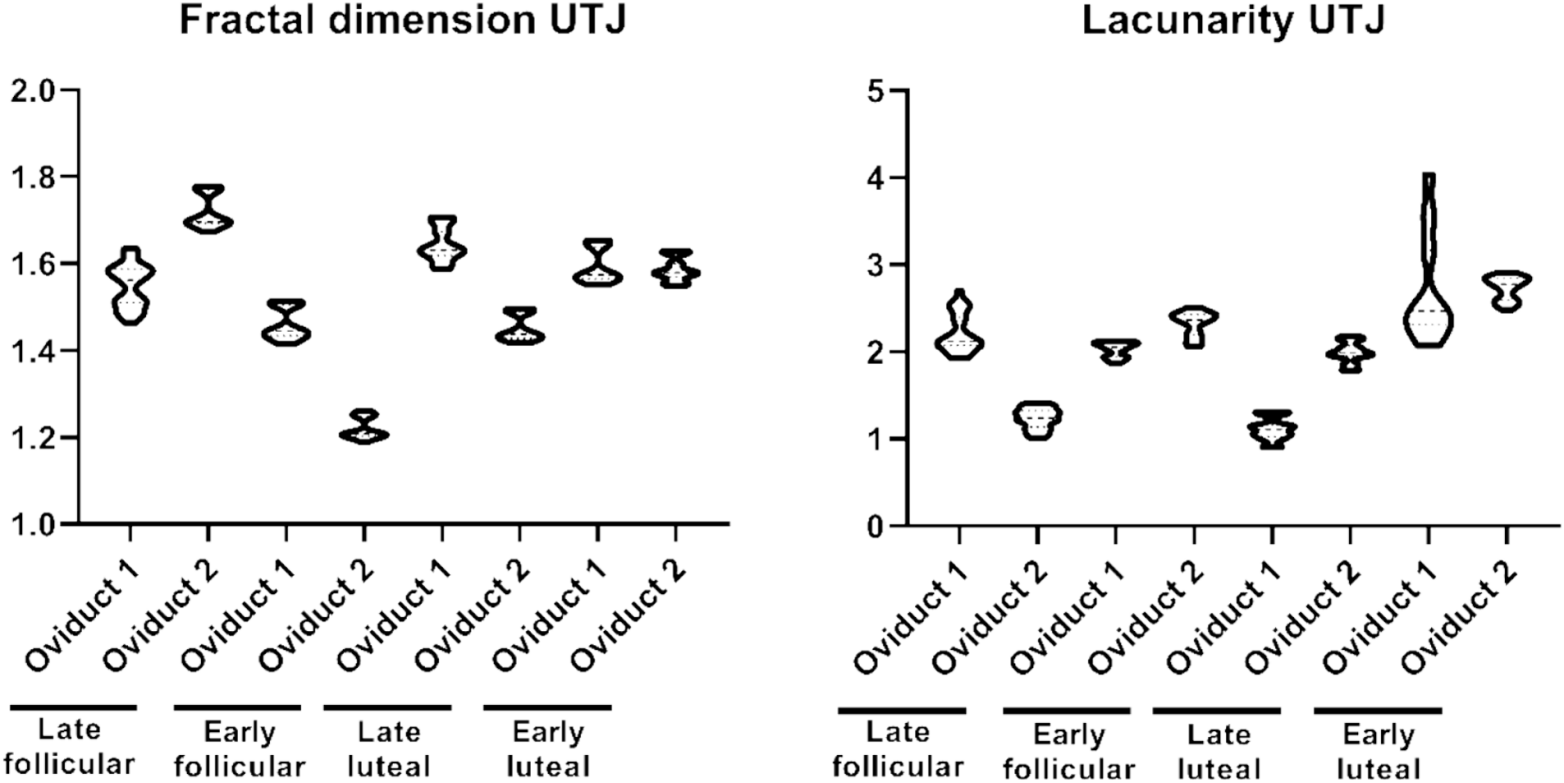
Violin plot representing the different measurement of fractal dimension (left) and lacunarity (right) of the folds from the UTJ.

On the contrary, the distribution of values for fractal dimension and lacunarity in UTJ is concentrated around a different subpopulation (Figure 2). The fractal dimension encompasses values within the range 1.189–1.1779, while the lacunarity values range within 0.901–2.701 (Supplementary Table S1). On the other hand, the shape factor values of the UTJ external and internal regions are provided (Table 1).

**Table 1:** Shape factor of the oviduct in UTJ region.

External shape factor								
	Late follicular		Early follicular		Late luteal		Early luteal	
	Oviduct 1	Oviduct 2	Oviduct 1	Oviduct 2	Oviduct 1	Oviduct 2	Oviduct 1	Oviduct 2
Volume (mm^3^)	66.31	127.71	31.53	26.64	41.55	43.64	84.22	39.80
Surface (mm^2^)	234.11	353.68	124.53	109.56	170.13	130.69	268.53	143.59
Height (mm)	4.46	4.46	4.46	4.46	4.46	4.46	4.46	4.46
Shape index	15.75	12.35	17.62	18.34	18.26	13.36	14.22	16.09
shape index (1/mm)	3.53	2.77	3.95	4.11	4.09	2.99	3.19	3.61
**Internal shape factor**								
Volume (mm^3^)	3.95	3.2	0.52	0.80	1.66	1.58	7.78	2.87
Surface (mm^2^)	77.88	50.2	13.83	16.31	30.20	23.92	106.27	30.19
Height (mm)	4.46	4.46	4.46	4.46	4.46	4.46	4.46	4.46
Shape index	87.94	69.97	118.62	90.93	81.14	67.52	60.92	46.92
shape index (1/mm)	19.72	15.69	26.60	20.39	18.19	15.14	13.66	10.52

### 2.2 Ampullary-isthmic junction

**Figure 3:**
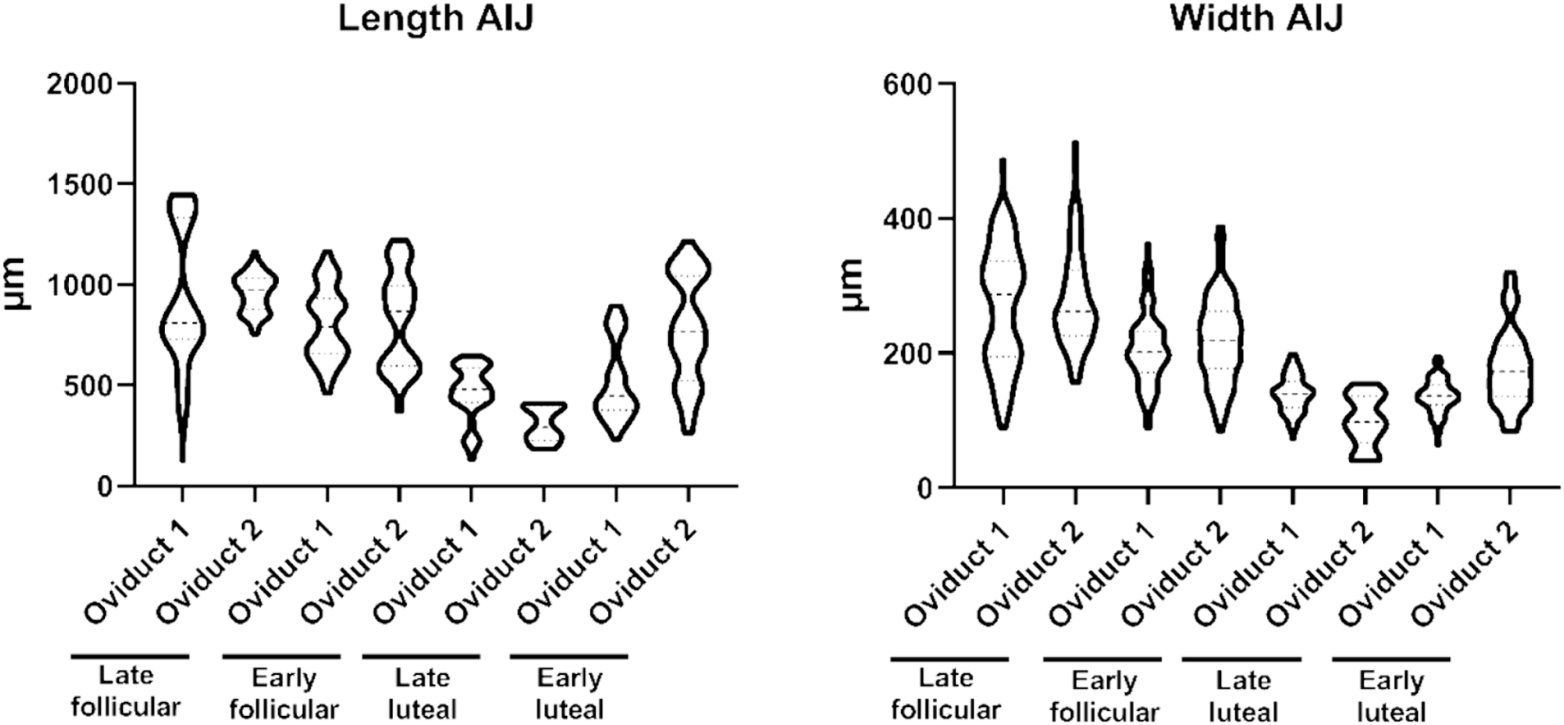
Violin plot representing the different measurement of length (left) and widths (right) of the folds from the AIJ.

In this section of the oviduct, we not only observe a higher prevalence of folds but also an increase in their individual sizes, ranging between 126–1,446 μm length (Supplementary Table S2) and 40–512 μm width (Supplementary Table S2), resulting in a more substantial occupancy within the lumen, reducing the lacunarity (Supplementary Table S2). In AIJ, distinct subpopulations of folds are discernible, with one set characterized by larger dimensions and another set exhibiting smaller dimensions. By contrast to the width measurements, where the data distribution is not centered around specific values, but rather displays a more homogeneous spread (Figure 3).

**Figure 4:**
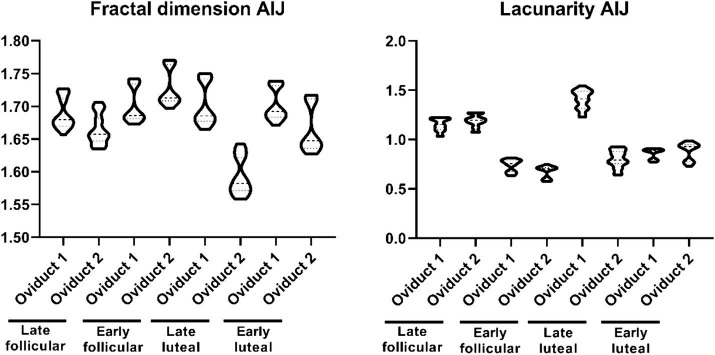
Violin plot representing the different measurement of fractal dimension (left) and lacunarity (right) of the folds from the UTJ.

Similar to what we observed with the length of the folds, the fractal dimension and lacunarity are also divided into different subpopulation (Figure 4). The fractal dimension encompasses values within the range 1.559–1.770, while the lacunarity values ranges within 0.577–1.544 (Supplementary Table S2). On the other hand, the shape factor values of the AIJ external and internal region is provided (Table 2).

**Table 2:** Shape factor of the oviduct in AIJ region.

External shape factor								
	Late follicular		Early follicular		Late luteal		Early luteal	
	Oviduct 1	Oviduct 2	Oviduct 1	Oviduct 2	Oviduct 1	Oviduct 2	Oviduct 1	Oviduct 2
Volume (mm^3^)	40.79	44	23.6	26.51	29.83	23.79	35.10	48.41
Surface (mm^2^)	226.97	225.73	234.99	286.61	155.14	84.67	166.89	323.40
Height (mm)	4.46	4.46	4.46	4.46	4.46	4.46	4.46	4.46
shape index	24.82	22.88	44.41	48.21	23.20	15.87	21.21	29.79
shape index (1/mm)	5.56	5.13	9.96	10.81	5.20	3.56	4.75	6.68
**Shape factor**								
Volume (mm^3^)	4.87	4.32	6.36	5.88	1.99	0.40	0.81	8.34
Surface (mm^2^)	66.76	73.22	103.86	141.18	41.52	8.96	18.91	195.45
Height (mm)	4.46	4.46	4.46	4.46	4.46	4.46	4.46	4.46
shape index	61.15	75.59	72.83	107.16	92.96	99.90	104.12	104.56
shape index (1/mm)	13.71	16.95	16.33	24.03	20.84	22.40	23.35	23.44

### 2.3 3-D reconstruction

**Figure 5:**
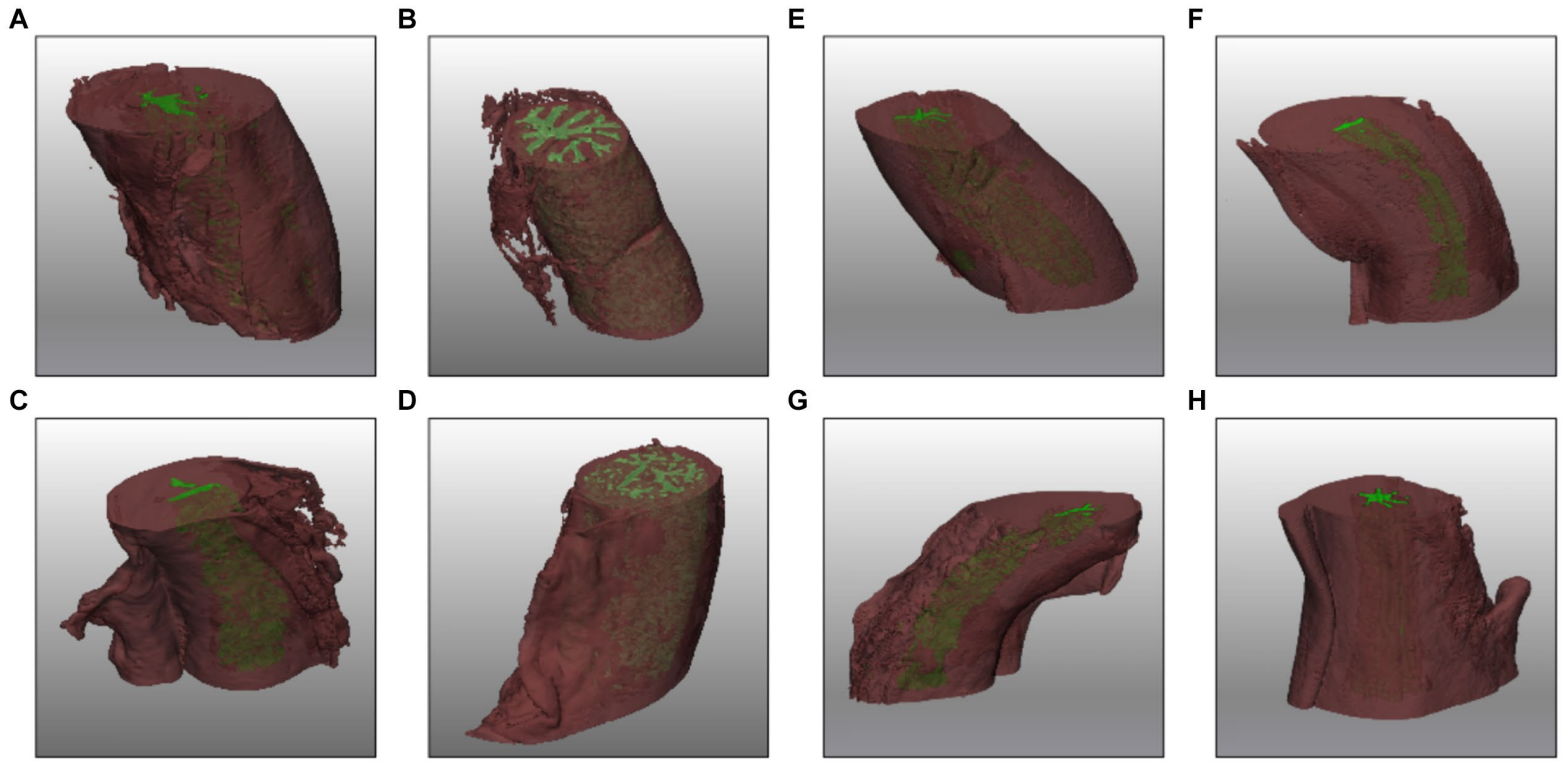
3D reconstruction of AIJ **(A–D)** and UTJ **(E–H)** regions in different phases of the cycle late follicular **(A,E)**, early follicular **(B,F)**, late luteal **(C,G)**, and early luteal **(D,H)**.

In the 3D reconstruction, the tortuous structure of the oviduct is represented offering a detailed view of the high tortuosity it possesses (Supplementary Movie 1). The visualization highlights the spatial arrangement of the folds of the AIJ section and small blind-ended sacs (Figures 5A–D). On the other hand, in the UTJ of the oviduct, the lumen is narrower and exhibits a less tortuous structure compared to the other sections (Figures 5E–H).

## 3 Discussion

In this study, we have delved into the architectural examination of different segments of swine oviduct by using a medical imaging approach, reporting for the first-time parameters such as the shape factor, fractal dimension and lacunarity of the oviduct through microCT imaging. These parameters will be the basis for a more accurate representation of reality in an oviduct-on-a-chip, enabling improved IVF and embryo culture performance, presumably enhancing epigenetic embryo reprogramming (9).

MicroCT has already been used to characterize the architecture of organs such as lungs (12), tendons or arteries (13), or even to recreate 3D images of breast cancer specimens (14). It generates 3D models that can then be converted into files suitable for 3D printing, thus enabling the creation of three-dimensional replicas of those organs (15). This technology has been used to create custom prostheses (16), and could be used to create models for surgeon planning (17) or for educational purposes (18). Recently, it has been used to create 3D bone phantoms through 3D printing, using CT generated images (19). In this context, this preliminary study means a progress into the creation of a novel oviduct 3D model considering the architecture of the organ, in the light to design a biomimetic 3D scaffold suitable for IVP.

The classical method for reconstructing the oviduct has traditionally involved using histology slides. However, it has been shown that this method may not be the most suitable, as it could result in gaps (20). Additionally, when using histology, it can be reconstruction inaccuracies ranging due to deformation of misalignment (21), due to the difficulty of managing three-dimensional orientation. Multiple manual artifacts affect the perfect alignment of the sample in all directions of space. In fact, the positioning procedures of the sample inside the embedding medium, the mounting of the histological specimen on the microtome, the simultaneous three-dimensional angulation of the specimen and the instrument are evident causes of problematic management of the spatial orientation of the specimens. This type of problem is partially overcome in the case of virtual histology that can be performed by microCT, which also becomes the basis of the three-dimensional model perfectly fitting the original sample. MicroCT technique has proven to be a valuable tool for studying mineralized biologic tissues (22), although its utility in the investigation of soft tissues has been somewhat constrained due to the limited contrast these tissues typically exhibit (23). Some tries have been made previously to study the internal structure of the oviduct through microCT (24). With the oviduct unmodified and without contrast, and other ones fill the oviduct with a strong contrast agent (24). The initial attempt proved to be unsuccessful as it yielded no discernible internal structures. In contrast, the subsequent approaches provided what could be likened to a photographic “negative” of the oviduct. This effect was achieved due to the significant disparity in clarity between the contrast agent and the surrounding tissue. However, when compared with histology slides, the structure observed was not very detailed. In our work, we employed the paraffin embedding method, a technique that enables us to visualize the high-resolution structure of the oviduct by leveraging its endogenous contrast, as previously described for murine embryos (25). Although in human the use of MicroCT to study the oviduct has been already achieved successfully (26), to our knowledge, those represent the first data obtained from swine oviducts through microCT.

Like what has been observed in humans (26, 27) or sheep (28), our results show throughout all phases of the cycle, at the UTJ level, mucosal folds are sparse and smaller in size, occupying limited space within the lumen. However, as we move away from the uterus and progress toward the oviduct until the ampulla, a significant increase in the size of these folds and the proportion of space they occupy within the lumen becomes evident. This increase in lumen occupancy is not only apparent from the measurements of the folds in the images but is also corroborated by the decrease in lacunarity. In addition, even though our sample size is small, it is worth noting that the maximum fold amplitude is consistently observed during the follicular phase, as previously described using electronic microscopy (29). Cyclic variations during the estrous cycle have been even described in the serosal part of the oviduct where cells of the tubal epithelium are also present, being the ciliated cells predominant during the follicular phase and the secretory cells during the luteal phase (30). It has been suggested that the folds in the oviduct could play a crucial role in the transport of oocytes and embryos during the fertilization process. These oviductal epithelial folds serve to significantly increase the surface area of the epithelium, thereby enhancing the likelihood of contact between the oocyte/embryo and the ciliated cells within the oviduct (31). Moreover, the specific structure of these folds appears to play a key role in alleviating the pressure difference in the oviductal fluid before and after the passage of the oocyte/embryo (31). It has been observed that the Celsr1 gene controls the proper formation of these oviductal folds, and Celsr1-deficient mice present an altered ciliary beating coordination, and aberrant fold orientation distribution, even leading to infertility (32), presumably due to difficulties in the effective transport of oocytes and embryos through the oviduct.

In the 3D reconstruction of the oviduct, we can observe numerous blind sacs or crypts, which are small, pouch-like structures within the oviduct. These anatomical features have been described in pigs (29) and in other animal species, including humans (26), ovine (28), marsupials (33, 34), hamster (35), bovine (6), and moles (36). In some animals, such as shrews, it has been suggested that the crypts in the oviduct mucosa may serve the function of “sequestering” sperm, thereby preventing polyspermy (37). Meanwhile, in other animals like cows (6) or sows (29), it has been proposed that these structures could also collaborate to form the sperm reservoir.

The data gleaned from this study possesses significant potential to lay the groundwork for the development of a sophisticated 3D model. Advanced printing techniques, including additive manufacturing processes, can then be employed to fabricate the physical device layer by layer, resulting in a tangible replica of the organ. This model would be meticulously crafted to encompass the intricate architecture of the oviduct. By meticulously incorporating the detailed structural nuances revealed by the microCT imaging, this 3D printing file would serve as a blueprint for creating a physical device that faithfully replicates the natural features of the organ. This innovative device, when combined with microfluidic systems, could have the potential to replicate the physiology of organs, offering a valuable tool for enhanced studies of organ function and disease (38). Notably, in recent years, research has shown that conducting *in vitro* fertilization processes within devices mimicking the oviduct can yield superior outcomes (9, 39). Considering this, the reconstructions obtained in this study hold the promise of serving as the blueprint for a 3D-printed device that accurately reflects this complex architecture. Thanks to its accuracy anatomy reconstruction, together with a microfluidic system, could even allow the study of the biophysics of the organ and a more reliable representation of the processes that’s occurs in the oviduct, as sperm capacitation or the “taxis” (chemotaxis, thermotaxis and rheotaxis) that guides sperm cells within the oviduct (40).

In this work, we conducted a comprehensive assessment to determine the most suitable segmentation techniques for accurately representing the morphological traits of interest. This evaluation encompassed various segmentation methods, including automatic, interactive, and manual approaches. The challenge of segmentation in biomedical imaging, as highlighted in both the existing literature and this experimental investigation, predominantly arises from the lack of efficient automated tools.

In summary, the pursuit of optimal segmentation techniques in biomedical imaging is crucial for advancing both research and clinical practice. By addressing the challenges inherent in segmentation, such as automation and accuracy, researchers and clinicians can harness the full potential of biomedical imaging. Additionally, the integration of 3D printing technology further amplifies the impact of biomedical imaging by that facilitating the conversion of virtual models into physical prototypes capable of faithfully reproducing even the most intricate shapes and geometric features. For that reason and considering that our group have previously identified a suitable material together with the suitable appropriate 3D printing technology, the data presented in this work will allow the engineer of a new 3D scaffold as closely as possible to the oviduct in order to increase the quality of embryos produced with ARTs.

## 4 Materials and methods

### 4.1 Oviduct selection and collection

Genital tracts from sows and gilts were obtained at the local slaughterhouse and transported into the lab within 2 h of slaughter. Once in the lab, the cycle stage of the tracts was determined based on the ovarian morphology as described previously (41) and classified into early follicular (*n* = 2), late follicular (*n* = 2), early luteal (*n* = 2), or late luteal phase (*n* = 2). Two oviduct from 4 animals were selected for each phase of the reproductive cycle, dissected and divided into segments. The portions corresponding to the isthmus and the uterine-tubal junction were washed in PBS and fixed in 4% paraformaldehyde for 1 h. Subsequently, the samples underwent dehydration through a series of alcoholic solutions (ranging from 50 to 100%) and soaked in xylene 3 times for 15 min each (45 min tot). An incubation step with xylene paraffin (1:1) was carried out for 45 min at 56°C before embedding in paraffin wax.

### 4.2 MicroCT and image acquisition

MicroCT datasets of swine oviducts at different stages of estrous cycle were acquired by using the high-resolution 3D-imaging system Skyscan 1172G (Bruker, Kontich – Belgium), using an L7901-20 Microfocus X-ray Source (Hamamatsu), with image pixel/size of 7.4 μm, camera binning 2×2, source voltage of 39 kV, source current of 240 μA, exposure time of 500 ms. The reconstructed tomographic volumes of the acquired images were performed using built-in NRecon Skyscan reconstruction software (Version: 1.6.6.0; Skyscan Bruker). 3D-images were generated using 3D-Visualization Software CTvox v. 2.5, while the volume rendering and virtual sectioning views using DataViewer v. 1.4.4 (Skyscan Bruker) and the analysis of the sample was performed using CT-Analyser software version 1.13.

### 4.3 Histology and light microscopy

**Figure 6:**
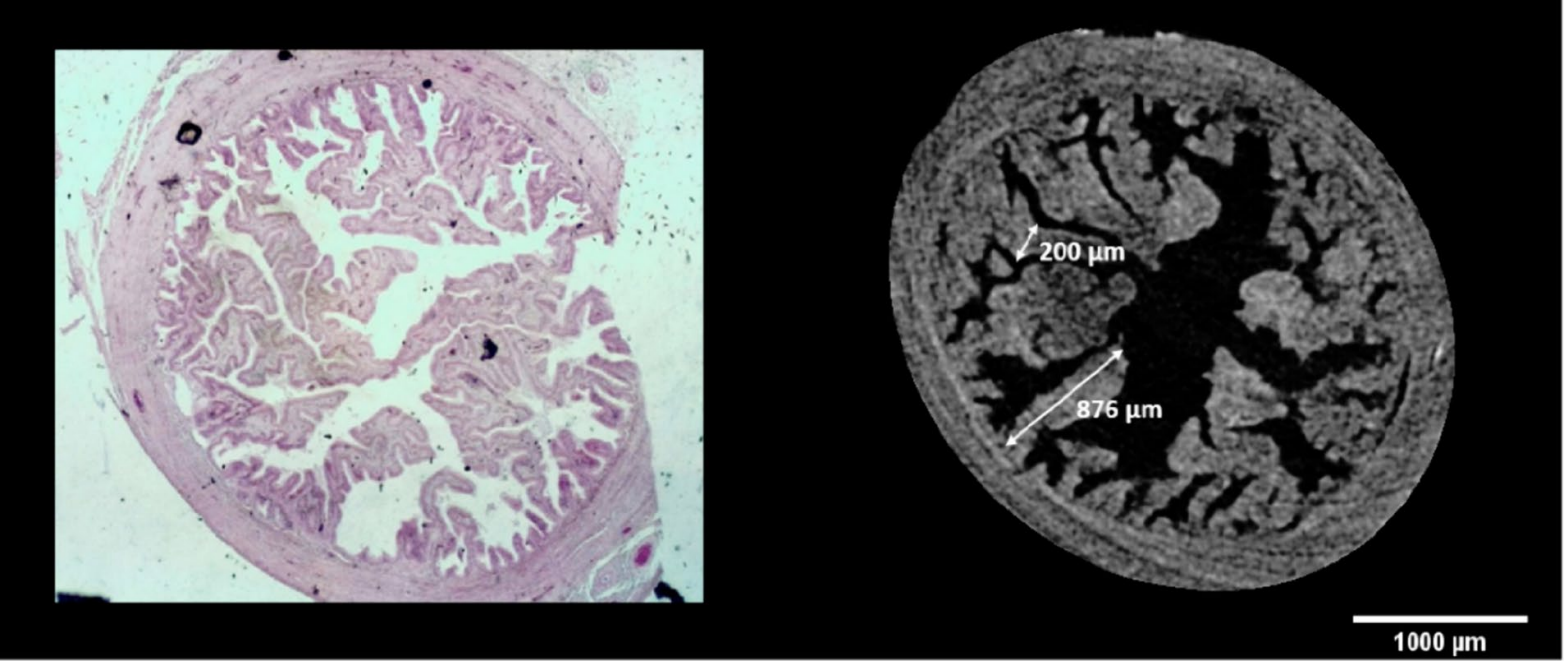
Comparison between virtual and histological oviduct sectioning, with a schematic representation of the measurement method performed.

Paraffin embedded oviducts were microtome-sectioned at 10 μm and stained with Hematoxylin (Sigma-Aldrich, cat. MHS16) – Eosin (Sigma-Aldrich, cat. 109,844) standard protocol to perform histological assay. Images were obtained with the stereomicroscope MZ12 (Leica) equipped with a color camera. The histological analysis highlighted the accuracy and fidelity of the two-dimensional and three-dimensional images obtained from microtomography, which has the further advantage of guaranteeing the structural integrity of the sample and avoiding distortions and artifacts resulting from the sectioning procedures (Figures 2, 5, 6).

### 4.4 Image analysis

Using microCT images, measurements of the length and width of the oviduct folds were conducted, as illustrated in Figure 6. The FIJI program (ImageJ 2.0.0-rc-43/1.50e) was employed for this purpose, utilizing its built-in measurement tool to ensure accuracy in anatomical dimensions. Similarly, to calculate the fractal dimension and lacunarity values, we applied the box-counting method using the FracLac plugin in Fiji.

### 4.5 Two-dimensional image processing

For the processing of two-dimensional images, the software Mimics from the Belgian company Materialise (Materialise, Leuven, Belgium) and 3Matic (Materialise, Leuven, Belgium) were used. Mimics is the primary software for processing biomedical images in the rapid prototyping sector. Initially, the DICOM files were loaded, and the correct orientation of the images (Top-Bottom, Anterior–Posterior, Right–Left) was set. Once the images were loaded, the software provided their visualization of the three anatomical planes: transverse/axial, coronal/frontal, and sagittal. It was decided to analyze, in particular, the series of images from the ampullary-isthmus junction (AIJ) and of the utero-tubal junction (UTJ). This approach ensured that images from all phases acquired were available, regardless of their original format. Each image series generated a project within Mimics, i.e., a file in .mcs format. At this point, an initial segmentation was performed on each of the four files by selecting the region of interest through the crop project operation. Specifically, various slices along all three directions (x, y, z) were excluded from the project, retaining only the structures of interest and reducing the number of slices to be analyzed. This helped eliminate the influence of regions not relevant to the study. To achieve this, the position and size of a rectangle were set in all three views (axial, coronal, and sagittal) to delimit the volume of interest in the resulting parallelepiped in the three-dimensional view. After a careful analysis of the images from various contrast phases, the different anatomical structures that would be included in the overall three-dimensional virtual model have been segmented separately: both the external and internal structures of the reference tract (UTJ and AIJ). For each of these structures, it was assessed which image series was most suitable for their segmentation. This decision was based on the presence of enhanced visibility of the mentioned structures in those specific images. Regarding the improvement of image quality, especially in terms of contrast, a point-wise enhancement technique was employed. This involves applying a transformation that maps a small range of grey levels onto the entire possible range, aiming to achieve visual enhancement. Finally, it is specified that the term “mask” refers to a set of pixels that have been grouped together as a result of various operations performed on the three two-dimensional views. Each mask is associated with a specific color, a minimum HU value, and a maximum HU value.

### 4.6 Processing of the three-dimensional model

Once the segmentation of the different structures has been carried out, it is necessary to process the three-dimensional model by performing a smoothing operation with the appropriate number of iterations. This is done to achieve a virtual model suitable for both three-dimensional visualization and the subsequent potential 3D printing phase. The three-dimensional object calculated from the mask inevitably exhibits a step effect due to the spatial resolution of the images, which is evidently insufficient for our purposes. The edges of the three-dimensional object, reconstructed from the mask, tend to follow individual pixels that have dimensions of approximately 1 mm × 1 mm. This scaling effect can be observed both in the three-dimensional view and on the individual slices for each anatomical structure. The smoothing operation was performed in the Mimics software rather than the modelling software 3-Matic by Materialise, used in the later phase of processing the three-dimensional model and optimizing the mesh. This choice is because, once the smoothing operation with a certain number of iterations is completed in Mimics, it is possible to compare the result with CT images on individual sections (axial, coronal, and sagittal) by visualizing the contours of the obtained three-dimensional object. In general, smoothing operations contribute significantly to enhancing the surface quality of a model. Nevertheless, it is crucial to exercise caution, as an excessively high number of smoothing iterations can lead to alterations in the model, resulting in unexpected outcomes. It is important to keep in mind that real-life organs do not possess perfectly smooth surfaces. The complete virtual model obtained in this way enabled the three-dimensional visualization of various anatomical structures. In this visualization, appropriate degrees of transparency were set for different structures, allowing for the visualization of outer and inner structures as well.

### 4.7 Processing of the three-dimensional model

The calculation of the shape factor is a procedure used in various contexts, such as physics, engineering, or thermodynamics. The shape factor is a quantity that expresses the geometry of one body in relation to another, often concerning thermal radiation exchange or fluid dynamics. Its mathematical expression can vary depending on the specific context.

In general terms, the shape factor (F) that we used can be calculated using the following simplified formula:


$$F=\frac{S*h}{V}$$

where S and V are, respectively, the surfaces and volumes of the two bodies under consideration, the UTJ and AIJ, and h is the height of the traits. However, in more complex situations, such as radiation exchange between non-ideal surfaces, the formula can be more intricate and involve angles, distances, and emissivity properties of the surfaces.

## Data Availability

The original contributions presented in the study are included in the article/Supplementary material, further inquiries can be directed to the corresponding author.

## References

[1] Mahé C Zlotkowska AM Reynaud K Tsikis G Mermillod P Druart X. Sperm migration, selection, survival, and fertilizing ability in the mammalian oviduct†. Biol Reprod. (2021) 105:317–31. doi: 10.1093/biolre/ioab105, PMID: 34057175 PMC8335357

[2] Hugentobler SA Sreenan JM Humpherson PG Leese HJ Diskin MG Morris DG. Effects of changes in the concentration of systemic progesterone on ions, amino acids and energy substrates in cattle oviduct and uterine fluid and blood. Reprod Fertil Dev. (2010) 22:684–94. doi: 10.1071/RD09129, PMID: 20353728

[3] Ménézo Y Guérin P Elder K. The oviduct: a neglected organ due for re-assessment in IVF. Reprod Biomed Online. (2015) 30:233–40. doi: 10.1016/j.rbmo.2014.11.011, PMID: 25599823

[4] Talevi R Gualtieri R. Molecules involved in sperm-oviduct adhesion and release. Theriogenology. (2010) 73:796–801. doi: 10.1016/j.theriogenology.2009.07.005, PMID: 19682733

[5] Steinhauer N Boos A Günzel-Apel AR. Morphological changes and proliferative activity in the oviductal epithelium during hormonally defined stages of the oestrous cycle in the bitch. Reprod Domest Anim. (2004) 39:110–9. doi: 10.1111/j.1439-0531.2004.00490.x, PMID: 15065993

[6] Yániz JL Lopez-Gatius F Santolaria P Mullins AKJ. Study of the functional anatomy of bovine Oviductal mucosa. Anat Rec. (2000) 260:268–78. doi: 10.1002/1097-0185(20001101)260:3<268::AID-AR60>3.0.CO;2-L, PMID: 11066037

[7] Abe H. The mammalian oviductal epithelium: regional variations in cytological and functional aspects of the oviductal secretory cells. Histol Histopathol. (1996) 11:743–68. PMID: 8839764

[8] Canovas S Ivanova E Romar R García-Martínez S Soriano-Úbeda C García-Vázquez FA. DNA methylation and gene expression changes derived from assisted reproductive technologies can be decreased by reproductive fluids. eLife. (2017) 6:23670. doi: 10.7554/eLife.23670, PMID: 28134613 PMC5340525

[9] Ferraz MAMM Rho HS Hemerich D Henning HHW van Tol HTA Hölker M. An oviduct-on-a-chip provides an enhanced *in vitro* environment for zygote genome reprogramming. Nat Commun. (2018) 9:9. doi: 10.1038/s41467-018-07119-8 30467383 PMC6250703

[10] Michael J Modell H McFarland J Cliff W. The “core principles” of physiology: what should students understand? Adv Physiol Educ. (2009) 33:10–6. doi: 10.1152/advan.90139.2008 19261754

[11] Belda-Perez R Heras S Cimini C Romero-Aguirregomezcorta J Valbonetti L Colosimo A. Advancing bovine *in vitro* fertilization through 3D printing: the effect of the 3D printed materials. Front Bioeng Biotechnol. (2023) 11:1260886. doi: 10.3389/fbioe.2023.1260886 37929185 PMC10621798

[12] Scott AE Vasilescu DM Seal KAD Keyes SD Mavrogordato MN Hogg JC. Three dimensional imaging of paraffin embedded human lung tissue samples by micro-computed tomography. PLoS One. (2015) 10:e0126230. doi: 10.1371/journal.pone.0126230, PMID: 26030902 PMC4452358

[13] Shearer T Bradley RS Hidalgo-Bastida LA Sherratt MJ Cartmell SH. Three-dimensional visualisation of soft biological structures by X-ray computed micro-tomography. J Cell Sci. (2016) 129:2483–92. doi: 10.1242/jcs.179077, PMID: 27278017

[14] DiCorpo D Tiwari A Tang R Griffin M Aftreth O Bautista P. The role of Micro-CT in imaging breast cancer specimens. Breast Cancer Res Treat. (2020) 180:343–57. doi: 10.1007/s10549-020-05547-z, PMID: 32020431

[15] Eltorai AEM Nguyen E Daniels AH. Three-dimensional printing in orthopedic surgery. Orthopedics. (2015) 38:684–7. doi: 10.3928/01477447-20151016-05 26558661

[16] Dai KR Yan MN Zhu ZA Sun YH. Computer-aided custom-made hemipelvic prosthesis used in extensive pelvic lesions. J Arthroplast. (2007) 22:981–6. doi: 10.1016/j.arth.2007.05.002, PMID: 17920469

[17] Starosolski ZA Kan JH Rosenfeld SD Krishnamurthy R Annapragada A. Application of 3-D printing (rapid prototyping) for creating physical models of pediatric orthopedic disorders. Pediatr Radiol. (2014) 44:216–21. doi: 10.1007/s00247-013-2788-9, PMID: 24202430

[18] Shelmerdine SC Simcock IC Hutchinson JC Aughwane R Melbourne A Nikitichev DI. 3D printing from microfocus computed tomography (micro-CT) in human specimens: education and future implications. Br J Radiol. (2018) 91:1–8. doi: 10.1259/BJR.20180306 PMC6209478 29698059

[19] Mei K Pasyar P Geagan M Liu LP Shapira N Gang GJ. Design and fabrication of 3D-printed patient-specific soft tissue and bone phantoms for CT imaging. Res Sq. (2023) 13:17495. doi: 10.1038/s41598-023-44602-9, PMID: 37840044 PMC10577126

[20] Senter-Zapata M Patel K Bautista PA Griffin M Michaelson J Yagi Y. The role of Micro-CT in 3D histology imaging. Pathobiology. (2016) 83:140–7. doi: 10.1159/000442387 27100885

[21] Gibson E Gaed M Gómez JA Moussa M Romagnoli C Pautler S. 3D prostate histology reconstruction: an evaluation of image-based and fiducial-based algorithms. Med Phys. (2013) 40. doi: 10.1118/1.4816946 24007184

[22] Neues F Epple M. X-ray microcomputer tomography for the study of biomineralized endo- and exoskeletons of animals. Chem Rev. (2008) 108:4734–41. doi: 10.1021/cr078250m, PMID: 18754688

[23] Metscher BD. MicroCT for comparative morphology: simple staining methods allow high-contrast 3D imaging of diverse non-mineralized animal tissues. BMC Physiol. (2009) 9:11. doi: 10.1186/1472-6793-9-11, PMID: 19545439 PMC2717911

[24] Burkitt M Walker D Romano DM Fazeli A. Computational modelling of maternal interactions with spermatozoa: potentials and prospects. Reprod Fertil Dev. (2011) 23:976–89. doi: 10.1071/RD11032 22127003

[25] Ermakova O Orsini T Gambadoro A Chiani F Tocchini-Valentini GP. Three-dimensional microCT imaging of murine embryonic development from immediate post-implantation to organogenesis: application for phenotyping analysis of early embryonic lethality in mutant animals. Mamm Genome. (2018) 29:245–59. doi: 10.1007/s00335-017-9723-6, PMID: 29170794 PMC5887010

[26] Castro PT Aranda OL Matos APP Marchiori E de Araújo LFB Alves HDL. The human endosalpinx: anatomical three-dimensional study and reconstruction using confocal microtomography. Pol J Radiol. (2019) 84:e281–8. doi: 10.5114/pjr.2019.86824, PMID: 31482002 PMC6717942

[27] Rocca M El Habashy M Nayel S Madwar A. The intramural segment and the uterotubal junction: an anatomic and histologic study. Int J Gynaecol Obstet. (1989) 28:343–9. doi: 10.1016/0020-7292(89)90606-1 2565256

[28] Yániz JL Carretero T Recreo P Arceiz E Santolaria P. Three-dimensional architecture of the ovine oviductal mucosa. Anat Histol Embryol. (2014) 43:331–40. doi: 10.1111/ahe.12078 23848134

[29] Yaniz JL Lopez-Gatius F Hunter RHF. Scanning electron microscopic study of the functional anatomy of the porcine oviductal mucosa. J. Vet. Med. Series C. (2006) 35:28–34. doi: 10.1111/j.1439-0264.2005.00634.x, PMID: 16433670

[30] Yániz JL Recreo P Carretero T Arceiz E Hunter RHF López-Gatius F. The peritoneal mesothelium covering the genital tract and its ligaments in the female pig shows signs of active function. Anat Rec. (2007) 290:831–7. doi: 10.1002/ar.20554 17538982

[31] Koyama H Shi D Fujimori T. Biophysics in oviduct: planar cell polarity, cilia, epithelial fold and tube morphogenesis, egg dynamics. Biophys Physicobiol. (2019) 16:89–107. doi: 10.2142/biophysico.16.0_89, PMID: 30923666 PMC6435019

[32] Shi D Komatsu K Hirao M Toyooka Y Koyama H Tissir F. Celsr1 is required for the generation of polarity at multiple levels of the mouse oviduct. Development. (2014) 141:4558–68. doi: 10.1242/dev.115659 25406397

[33] Bedford JM Breed WG. Regulated storage and subsequent transformation of spermatozoa in the fallopian tubes of an Australian marsupial, *Sminthopsis crassicaudata*. Biol Reprod. (1994) 50:845–54. doi: 10.1095/biolreprod50.4.845, PMID: 8199265

[34] Rodger JC Bedford JM. Induction of oestrus, recovery of gametes, and the timing of fertilization events in the opossum, *Didelphis virginiana*. J Reprod Fertil. (1982) 64:159–69. doi: 10.1530/jrf.0.0640159 7198686

[35] Smith TT Koyanagi F Yanagimachi R. Distribution and number of spermatozoa in the oviduct of the Golden Hamster after natural mating and artificial lnseminatio&. Biol Reprod. (1987) 37:5–234. 10.1095/biolreprod37.1.225 3651547

[36] Bedford JM Mock OB Nagdas SK Winfrey VP Olson GE. Reproductive features of the eastern mole (*Scalopus aquaticus*) and star-nose mole (*Condylura cristata*). J Reprod Fertil. (1999) 117:345–53. doi: 10.1530/jrf.0.1170345 10690203

[37] Bedford JM Mock OB Phillips DM. Unusual ampullary sperm crypts, and behavior and role of the cumulus oophorus, in the oviduct of the least shrew, *Cryptotis parva*. Biol Reprod. (1997) 56:1255–67. doi: 10.1095/biolreprod56.5.1255, PMID: 9160726

[38] Huh D Hamilton GA Ingber DE. From three-dimensional cell culture to organs-on-chips. Trends Cell Biol. (2011) 21:745–54. doi: 10.1016/j.tcb.2011.09.005, PMID: 22033488 PMC4386065

[39] Ferraz MAMM Henning HHW Costa PF Malda J Melchels FP Wubbolts R. Improved bovine embryo production in an oviduct-on-a-chip system: prevention of poly-spermic fertilization and parthenogenic activation. Lab Chip. (2017) 17:905–16. doi: 10.1039/C6LC01566B 28194463

[40] Monteiro Melo Ferraz M d A Ferronato GDA. Opportunities involving microfluidics and 3D culture systems to the *in vitro* embryo production. Anim Reprod. (2023) 20:58. doi: 10.1590/1984-3143-ar2023-0058 PMC10449241 37638255

[41] Carrasco LC Romar R Avilés M Gadea J Coy P. Determination of glycosidase activity in porcine oviductal fluid at the different phases of the estrous cycle. Reproduction. (2008) 136: 833–842. doi: 10.1530/REP-08-0221 18753246

