# Supplementary material for: Exploring swine oviduct anatomy through micro-computed tomography: a 3D modeling perspective
Source: Front Vet Sci. 2024 Sep 3;11:1456524. doi: 10.3389/fvets.2024.1456524 (PMC11405376; doi:10.3389/fvets.2024.1456524)
Supplement: Supplementary file 2 [file Table_1.pdf]

Supplementary Table 1. Measurements of oviduct folds length and width (µm) in the UTJ region.

	Late follicular		Early Follicular		Late luteal		Early luteal	
	**Oviduct 1**	**Oviduct 2**	**Oviduct 1**	**Oviduct 2**	**Oviduct 1**	**Oviduct 2**	**Oviduct 1**	**Oviduct 2**
**Length (µm)**								
Median	585.6	369.2	369.2	401.9	332.7	760.9	407.3	444.1
10%	457.0	313.4	313.4	288.2	234.6	639.5	322.0	351.1
Min	410,7	289.0	289.0	241.9	143.88	466.0	143.7	319.0
90%	656.8	426.2	426.2	439.7	436.4	873.2	600.9	493.8
Max	737.1	461.5	461.5	469.0	492.2	987.6	812.9	549.4
**Width (µm)**								
Median	143.2	140.8	206.1	177.3	106.7	136.0	137.1	158.5
10%	114.0	110.2	116.1	115.9	80.0	96.99	93.24	103.8
Min	87.28	89.11	80.91	84.45	69.81	76.45	72.84	89.11
90%	195.0	172.8	255.2	221.9	154.7	182.9	178.2	263.3
Max	239.3	230.5	366.3	256.5	211.9	244.6	215.7	321.3
**Fractal dimension**								
Median	1.562	1.697	1.445	1.208	1.631	1.437	1.574	1.579
10%	1.480	1.683	1.421	1.197	1.594	1.422	1.556	1.553
Min	1.464	1.673	1.414	1.189	1.587	1.419	1.551	1.548
90%	1.623	1.777	1.511	1.256	1.706	1.495	1.652	1.627
Max	1.635	1.779	1.514	1.261	1.707	1.497	1.654	1.630
**Lacunarity**								
Median	2.120	1.238	2.052	2.365	1.109	1.987	2.467	2.774
10%	1.963	1.061	1.895	2.086	0.908	1.787	2.103	2.533
Min	1.928	1.012	1.865	2.061	0.901	1.783	2.071	2.483
90%	2.541	1.386	2.116	2.470	1.310	2.155	3.699	2.872
Max	2.704	1.417	2.127	2.510	1.316	2.181	4.039	2.906
