# Supplementary material for: Exploring swine oviduct anatomy through micro-computed tomography: a 3D modeling perspective
Source: Front Vet Sci. 2024 Sep 3;11:1456524. doi: 10.3389/fvets.2024.1456524 (PMC11405376; doi:10.3389/fvets.2024.1456524)
Supplement: Supplementary file 3 [file Table_2.pdf]

Supplementary Table 2. Measurements of oviduct folds length and width (µm) in the AIJ region.

	Late follicular		Early Follicular		Late luteal		Early luteal	
	**Oviduct 1**	**Oviduct 2**	**Oviduct 1**	**Oviduct 2**	**Oviduct 1**	**Oviduct 2**	**Oviduct 1**	**Oviduct 2**
**Length (µm)**								
Median	809.6	972.2	789.6	867.2	480.1	290.1	445.6	766.4
10%	504.9	838.5	584.1	543.0	222.6	189.9	319.5	427.0
Min	126.0	754.7	461.5	369.2	133.6	182.5	232.1	264.8
90%	1415	1072	1046	1154	616.8	407.1	818.5	1109
Max	1446	1161	1166	1220	647.5	410.8	894	1214
**Width (µm)**								
Median	287.3	261.8	201.6	218.5	139.3	97.94	136.4	172.7
10%	148.1	201.7	141.5	139.8	104.6	40.69	101.3	117.2
Min	89.11	156.8	89.42	84.37	73.34	40.02	64.13	84.37
90%	386.3	392.5	279.2	294.6	178.7	149.5	168.5	277.5
Max	486.9	512.7	362.3	387.8	198.8	155.0	193.8	320.3
**Fractal dimension**								
Median	1.679	1.657	1.686	1.713	1.686	1.582	1.692	1.647
10%	1.666	1.638	1.677	1.704	1.670	1.561	1.677	1.633
Min	1.656	1.635	1.673	1.697	1.665	1.559	1.671	1.628
90%	1.723	1.701	1.740	1.770	1.746	1.638	1.738	1.715
Max	1.727	1.707	1.742	1.770	1.750	1.642	1.739	1.717
**Lacunarity**								
Median	1.152	1.195	0.7559	0.7032	1.413	0.7915	0.8870	0.9286
10%	1.042	1.083	0.6469	0.5797	1.250	0.6545	0.7792	0.7584
Min	1.032	1.073	0.6329	0.5770	1.228	0.6418	0.7700	0.7273
90%	1.220	1.270	0.7955	0.7250	1.505	0.9142	0.9007	0.9703
Max	1.223	1.274	0.8141	0.7507	1.544	0.9283	0.9077	0.9879
